# 235. EDP-323, a First-in-Class, Oral, RSV-Specific, Non-Nucleoside L-Protein Inhibitor Antiviral Rapidly Reduces Total RSV Symptoms, Lower Respiratory Tract RSV Symptoms and Viral Load After Human Viral Challenge

**DOI:** 10.1093/ofid/ofaf695.087

**Published:** 2026-01-11

**Authors:** John DeVincenzo, Alaa Ahmad, Shijie Chen, Brandon Londt, Alexander J Mann, Julie Mori, Andrew P Catchpole, Scott T Rottinghaus

**Affiliations:** Enanta Pharmaceuticals, Watertown, MA; Enanta Pharmaceuticals, Watertown, MA; Enanta Pharmaceuticals, Watertown, MA; hVivo, London, England, United Kingdom; hVIVO, London, England, United Kingdom; hVivo Services LTD, London, England, United Kingdom; hVivo Services Ltd., London, England, United Kingdom; Enanta Pharmaceuticals, Watertown, MA

## Abstract

**Background:**

RSV impacts large populations of vulnerable children and adults despite available prevention strategies. No effective RSV treatments exist. EDP-323, a first in class, potent, oral, non-nucleoside small molecule inhibitor of RSV polymerase (L-protein) is in development to treat RSV infections.

**Figure:**
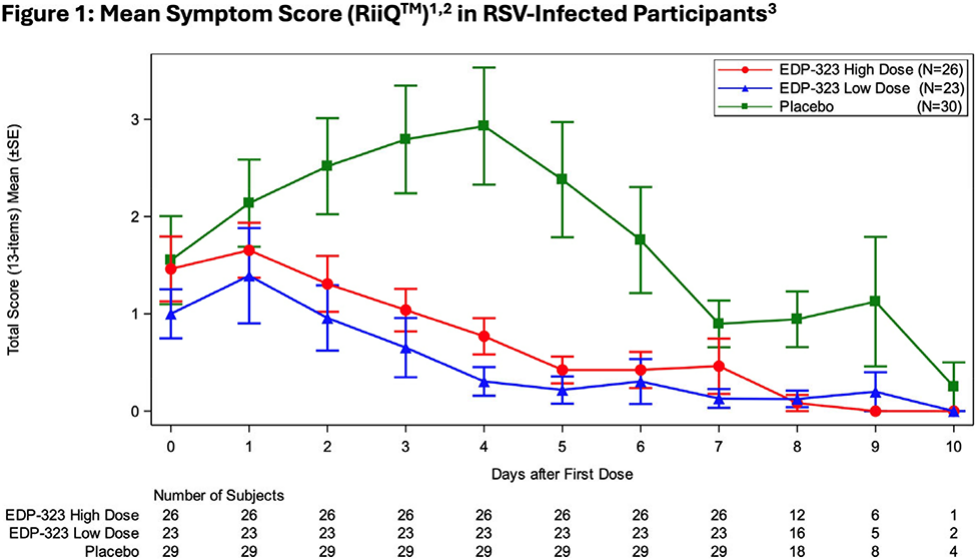

**Figure:**
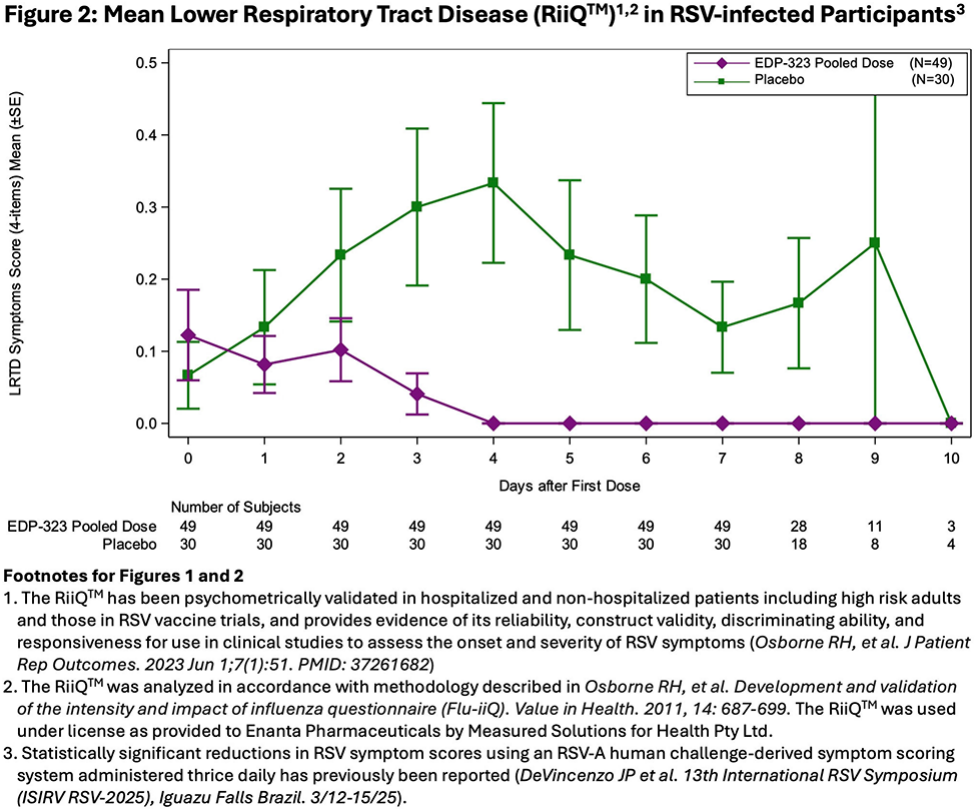

**Methods:**

A randomized, double-blind, placebo (PBO)-controlled study (NCT06170242) evaluated the efficacy, antiviral activity and safety, of EDP-323. Healthy volunteers were inoculated with RSV-A. After confirmed RSV infection or 5 days(D) later, randomized participants received EDP-323 600mg (n=47), 200mg (with 600mg loading dose; n=47), or PBO (n=47) once daily (QD) for 5D and were followed through 28D. Clinical symptoms were assessed QD using the Respiratory Infection Intensity and Impact Questionnaire (RiiQ™) and viral loads (VL) were assessed by qRT-PCR on nasal washes. We evaluated efficacy as area under the curve (AUC) effects in the intent-to-treat infected population (EDP-323 600mg [n=26]; 200mg [n=23]; PBO [n=30]).

**Results:**

Participants showed rapid (within the 1st 24 hr) and statistically significant improvements in RiiQ^TM^

RSV symptoms (Figs 1,2) and VL after EDP-323 dosing vs PBO. Compared to PBO, there were 73% (*P*=0.0012), 61% (*P*=0.0010), and 67% (*P*< 0.0001) RiiQ^TM^ total symptom score AUC reductions in 200mg, 600mg, and EDP-323 pooled recipients respectively. Lower respiratory tract disease scores (LRTD) AUC were reduced by 95% (*P*=0.0002), 73% (*P*=0.0088), and 85% (*P*=0.0002) respectively in the 200mg, 600mg, and Pooled EDP-323 recipients vs PBO. There were 87% and 85% VL AUC reductions in 200mg and 600mg recipients, respectively vs PBO (all *P*< 0.0001). EDP-323 dosing groups showed similar efficacies. Frequencies of treatment-emergent adverse events (TEAEs) were similar across EDP-323 and PBO groups. No serious TEAEs, severe AEs, or AEs leading to treatment discontinuation or study withdrawal occurred.

**Conclusion:**

EDP-323 rapidly and significantly reduced symptoms including lower respiratory tract symptoms as measured by the RiiQ^TM^ patient reported outcome tool, lowered viral load vs placebo in healthy RSV infected adults and was well-tolerated. These findings support EDP-323 as a potential once daily oral RSV treatment.

**Disclosures:**

All Authors: No reported disclosures

